# Salmon hatchery strays can demographically boost wild populations at the cost of diversity: quantitative genetic modelling of Alaska pink salmon

**DOI:** 10.1098/rsos.240455

**Published:** 2024-07-03

**Authors:** Samuel A. May, Kyle R. Shedd, Kristen M. Gruenthal, Jeffrey J. Hard, William D. Templin, Charles D. Waters, Milo D. Adkison, Eric J. Ward, Christopher Habicht, Lorna I. Wilson, Alex C. Wertheimer, Peter A. H. Westley

**Affiliations:** ^1^ College of Fisheries and Ocean Sciences, University of Alaska Fairbanks, Fairbanks, AK, USA; ^2^ Alaska Department of Fish & Game, Anchorage, AK, USA; ^3^ Alaska Department of Fish & Game, Juneau, AK, USA; ^4^ Conservation Biology Division, Northwest Fisheries Science Center, National Marine Fisheries Service, National Oceanic and Atmospheric Administration, Seattle, WA, USA; ^5^ Auke Bay Laboratories, Alaska Fisheries Science Center, National Marine Fisheries Service, National Oceanic and Atmospheric Administration Juneau, Juneau, AK, USA; ^6^ Fishheads Technical Services, Juneau, AK, USA

**Keywords:** hatchery–wild interactions, salmon aquaculture, eco-evolutionary dynamics, quantitative genetics, straying, portfolio effects

## Abstract

Hatcheries are vital to many salmon fisheries, with inherent risks and rewards. While hatcheries can increase the returns of adult fish, the demographic and evolutionary consequences for natural populations interacting with hatchery fish on spawning grounds remain unclear. This study examined the impacts of stray hatchery-origin pink salmon on natural population productivity and resilience. We explored temporal assortative mating dynamics using a quantitative genetic model that assumed the only difference between hatchery- and natural-origin adults was their return timing to natural spawning grounds. This model was parameterized with empirical data from an intensive multi-generational study of hatchery–wild interactions in the world’s largest pink salmon fisheries enhancement program located in Prince William Sound, Alaska. Across scenarios of increasing hatchery fish presence on spawning grounds, our findings underscore a trade-off between demographic enhancement and preservation of natural population diversity. While enhancement bolstered natural population sizes towards local carrying capacities, hatchery introgression reduced variation in adult return timing by up to 20%. Results indicated that hatchery-origin alleles can rapidly assimilate into natural populations, despite the reduced fitness of hatchery fish attributable to phenotypic mismatches. These findings elucidate the potential for long-term demographic and evolutionary consequences arising from specific hatchery–wild interactions, emphasizing the need for management strategies that balance demographic enhancement with the conservation of natural diversity.

## 1. Background

Salmon hatcheries around the world release captive-reared juvenile fish into the ocean, where they mature and migrate back to release sites to support commercial, recreational and subsistence fisheries. Hatcheries can also be used to mitigate or reverse declines in depleted populations [1,2]. However, concerns have risen over potential adverse ecological and genetic impacts of hatcheries on wild populations [3–7]. The impacts of large-scale fishery enhancement programs, intended to allow harvest segregation of hatchery and wild populations, are particularly controversial [1,8,9], in part because many unharvested hatchery-origin individuals may ‘stray’ onto the spawning grounds of wild populations [10,11]. Although straying occurs naturally among wild salmon populations [12], and hatchery strays are typically a small fraction of total returns, hatchery programs operating on scales of tens of millions of returning adults can contribute substantially to wild population demographics [11]. Therefore, understanding the intricate relationships between hatchery and wild fish populations is central to supporting socioeconomically important fisheries while conserving natural population diversity [1,4].

Hatchery-origin fish can differ from natural-origin fish in fitness-related traits, and strays may adversely affect the genetic and life history diversity of wild salmon populations [1,6]. Empirical studies report differences in fitness-linked traits between natural and hatchery-origin fish spawning in the wild [13,14], including morphological traits like reduced body size [15]; behavioural traits like aggression [16]; and reproductive traits like fecundity [16,17], reproductive lifespan (RLS; [17]), or survival and reproductive success (RS; [18–22]). Hatchery-origin salmon strays can shift allele frequencies of wild populations towards that of hatchery-origin fish through introgression, particularly when their life history traits are similar [7,23]. This can weaken the adaptive capacity of wild populations [24,25]. Straying can also alter life history diversity, including age at maturation and return timing [26–28], potentially disrupting wild temporal population dynamics through competition [16,17,29] or inducing phenological shifts [30].

Given the potential impacts on phenotypic and genetic diversity, concerns have arisen that hatcheries may weaken ecological portfolio effects [31,32]. Ecological portfolio effects suggest that phenotypic and life history diversity can buffer against environmental variability and changes in any one subunit [33,34]. In variable habitats, particularly those characterized by extinction and recolonization, increased asynchrony among connected population subunits enhances overall stability and resilience [35]. Resilience, in this context, refers to the capacity of populations or population complexes (i.e. metapopulations) to recover from perturbations and adapt to change, whereas stability refers to reduced variation in metapopulation size or genetic diversity [36]. Portfolio effects have been extensively studied in salmon systems, particularly associated with reduced variation in phenological traits like run timing and increased connectivity among populations [37–40]. Hatchery supplementation has reduced life history diversity in some natural salmon populations [28,31,32]; however, the underlying mechanisms and long-term consequences remain poorly understood. Thus, thorough investigations into the multi-faceted consequences of hatchery straying are needed, with complementary empirical and theoretical approaches to better understand underlying mechanisms.

The numerous possible interactions between hatchery and wild fish yield a range of outcomes from adverse effects to neutral or even beneficial impacts on wild salmonids [5,41,42]. Many studies report reduced relative reproductive success (RRS) of hatchery strays [13,14,21], although demographic consequences are contentious and often difficult to detect [28,43]. On the one hand, hatchery straying may decrease wild population fitness owing to the introgression of maladapted alleles or negative density dependence [44,45]. On the other hand, selection against hatchery lineages may purge deleterious alleles, leading to neutral or even positive impacts on wild populations owing to increased reproductive output [1,13,28,42,45]. These explanations are not mutually exclusive and likely depend on local fitness landscapes, context-specific hatchery–wild interactions, species life histories, hatchery straying magnitude and management practices [23,45,46]. Few studies examine hatchery straying impacts over multiple generations, resulting in uncertainty regarding the temporal consistency of effects. Additionally, studies providing insights are often from systems with confounded influences of habitat degradation [47], underscoring the need for careful studies investigating specific dynamics in specific ecological and genetic contexts. Collectively, these inconsistencies and knowledge gaps emphasize the importance of context-specific, longitudinal studies to comprehensively understand hatchery straying impacts on wild populations.

The timing of adult salmon return migrations is particularly important for fitness in salmonids via competition among adult spawners, impacts on juvenile development rate and timing of migration to sea [48]. Wild salmon phenology is strongly influenced by both genetic and environmental variation and local habitat characteristics [14,49,50]. Hatchery-origin fish may differ in return timing owing to broodstock selection practices, hatchery rearing conditions or juvenile release practices [51]. Many hatcheries select adult return timing to segregate hatchery from wild stocks, causing hatchery-origin strays to return at different, often less-variable times than wild counterparts [30,52,53]. For example, in Prince William Sound (PWS), Alaska, pink salmon (*Oncorhynchus gorbuscha*) broodstock selection favoured supratidal, later-returning fish, because of compatibility of the development rate, temperature regime of the local stream that provided hatchery rearing water and emergence timing [54,55]. However, underlying mechanisms driving lasting differences and ultimate effects on wild productivity and resilience remain controversial [1,51].

Pink salmon are an ideal species for studying hatchery–wild interactions owing to their unique 2 year life cycle and profound sociocultural and economic importance [8,9,11,56,57]. Spawning in functionally intact freshwater or intertidal habitats in PWS, the short lifespan of pink salmon facilitates multi-generational studies investigating long-term effects of introgression [21]. High productivity and stray rates compared to other salmonids facilitate the detection of strays in nature [12,58]. Distinct even- and odd-year broodlines also offer a natural comparative framework, lending confidence to behavioural and demographic trends seen across lineages. These features are exemplified in PWS, where an average of 600–700 million hatchery-origin juveniles have been released annually since 1988, representing the largest pink salmon hatchery program in the world [2,59]. From 1960 to 1976, before enhancement, PWS produced approximately 6–7 million pink salmon, with harvests of approximately 4 million. In contrast, between 2010 and 2019, harvest rose to approximately 50 million annually, over 80% of which was of hatchery-produced salmon [60]. While fisheries are managed to target a high harvest rate of hatchery-origin fish [61], large numbers of hatchery fish stray to wild spawning grounds [11,62], prompting concerns about repercussions for wild populations [8,57].

In response to these concerns, the Alaska Department of Fish and Game founded the Alaska Hatchery Research Program (AHRP) in collaboration with non-profit hatchery operators, the fishing industry and the University of Alaska. In 2011, the AHRP began a decade-long field sampling project of representative natural streams throughout PWS. Hundreds of thousands of salmon were sampled to reconstruct pedigrees with molecular methods [21], and otoliths were used to determine whether individuals originated in hatcheries or natural populations (otoliths in hatcheries were thermally marked). The present study uses previously published estimates of key population parameters from AHRP studies to parameterize simulation models. Notably, in PWS, most stocks of hatchery pink salmon return to streams up to a week after the mean of the wild runs [63,64], corresponding with the return timing of original donor stocks [54]. However, hatchery-origin fish have half as many adult offspring as wild counterparts, on average [21], with unknown consequences for population productivity and resilience. The systematic PWS hatchery releases, coupled with extensive historical and ongoing data, offer a unique opportunity to examine the mechanisms underlying hatchery impacts to genetic-, phenotypic- and population-level dynamics of natural systems.

This study sought to examine the effects of hatchery-origin pink salmon strays on wild population productivity and resilience, particularly given differences in return timing between hatchery and natural-origin adults (‘natural-origin’ referring to fish that were incubated in wild, not hatchery settings). Our objectives were to (i) investigate if empirically observed reduced RRS of hatchery-origin fish could be owing to return timing differences, (ii) analyse how the proportion of hatchery-origin spawners (pHOS) and their temporal segregation impacts natural salmon productivity, (iii) quantify the speed of hatchery-origin introgression into wild populations, and (iv) examine the effect of pHOS and temporal segregation on life history diversity. To achieve these objectives, we used simulation-based experiments within a quantitative genetic framework that captured temporal assortative mating dynamics. We parameterized simulations with data from the AHRP, which has intensively sampled pink salmon spawning streams in PWS across multiple generations [21]. Our findings are discussed in the context of pink salmon biology and fisheries enhancement in PWS but are broadly relevant to aquaculture impacts on wild populations across taxa.

## 2. Methods

### 2.1. Model overview and assumptions

**Figure 1.**
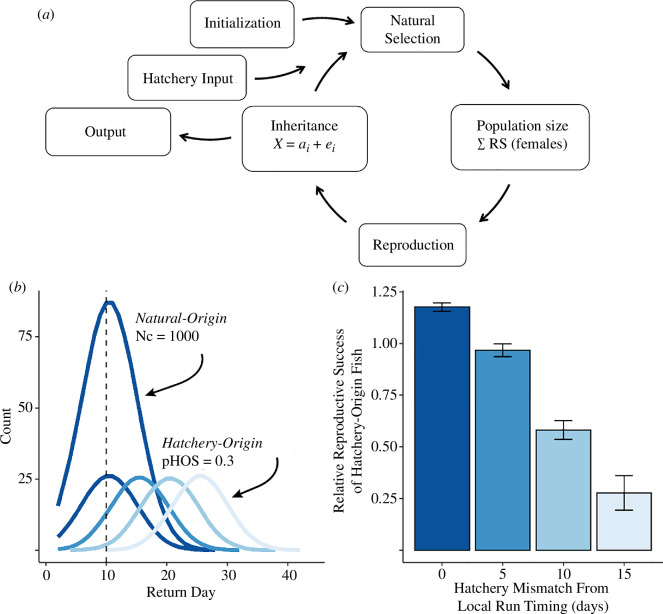
(*a*) Extended model workflow [65] with hatchery immigrants. (*b*) Return day distribution in simulated populations of 1000 natural fish and 4 hatchery-origin groups (colours). Dashed line indicates local phenotypic optimum and wild individual mean return day. (*c*) RRS (*y*-axis) of hatchery-origin groups versus temporal mismatch from local return timing (*x*-axis).

We extended a previously published individual-based model to forecast trait values and fitness across generations of simulated salmon populations [65]. Briefly, a population of adult natural-origin salmon of census size Nc_initial_ was initialized, and individuals were assigned trait values for return day to a simulated spawning stream and RLS in that stream, across a 45-day spawning season (figure 1). These traits were initially drawn from a bivariate Gaussian distribution, the shape of which was determined by a phenotypic variance–covariance matrix, which was an initial input parameter. Selection then acted on these traits via a bivariate, Gaussian fitness landscape to determine individual expected RS values, which were subsequently used to quantify the census size of the next generation (i.e. offspring recruitment). Offspring were assigned parents using a random draw of possible parent pairs, weighted by the product of the RRS of dams and sires. Whereas most quantitative genetic models assume random mating, a unique aspect of our framework is that it incorporates an assortative mating pattern; parent pairs must temporally overlap in the spawning stream to mate, based on the genetically and phenotypically correlated traits of return timing and lifespan in the stream. Offspring were then assigned trait values following quantitative genetic theory for deterministic inheritance: offspring trait values were drawn from a bivariate, Gaussian distribution with a mean equal to the midparent value and a variance and covariance set as initial input parameters. Offspring then became the parents of the next generation, and the model was run for a set number of generations. Interannual environmental variation was simulated by varying fitness landscape optima for return day and RLS after each time step, according to a predefined environmental variance parameter, such that populations constantly evolved towards the moving optima. Individual trait and fitness values within generations were used to quantify population-level summary statistics (table 1), such as census size and the mean and variance of traits, which were subsequently used to explore the evolution of traits and changes in population size over time.

**Table 1.** Descriptions of output parameters of interest examined in each of four modelling experiments, with input parameter values used in each experiment.

experiment	output parameter	description	pHOS	μ_Return day natural_	μ_Return day hatchery_
1	RRS	RRS of hatchery-origin fish	0.2	10	10, 15, 20, 25
2	Nc_Natural_	census population size of natural-origin fish	0.0, 0.01, 0.1, 0.3	10	15, 20, 25
3	pHOA	proportion hatchery origin ancestry in natural-origin fish, within cohorts	0.01, 0.1, 0.3	10	15, 20, 25
4	mean return day	mean return day of natural-origin individuals within cohorts	0.0, 0.1, 0.2, 0.3	5, 10, 13, 15, 17, 20	13

pHOA, proportion of hatchery-origin ancestry.

Model assumptions and detailed justifications for this framework are provided by May *et al*. [65]. Briefly, the model assumes a fixed spawning season duration, during which individuals return to streams, mate for a number of days and die; no spatial genetic or phenotypic structure within streams; balanced sex ratios with equal mating chances and equal trait distributions between sexes; constant phenotypic and genotypic variance–covariance matrices; inheritance via an infinitesimal model of many small-effect loci; and no additional intrinsic or extrinsic factors like predation, overwinter rearing conditions in fresh water, harvest, dispersal or inbreeding depression. This study added a density dependence fitness function, a cap on the number of unique mates for individuals and a hatchery-origin population with distinct phenotypes assumed to have strayed into simulated wild streams (details follow).

### 2.2. Density dependence module

In May *et al*. [65], simulated populations typically grew exponentially owing to the absence of density-dependent effects on mean fitness. Here, we included a density dependence function to better represent wild population dynamics and control population sizes. Empirical studies link higher spawner densities to increased pre-spawning mortality, subsequently reducing mean RS [66]. We addressed this by applying a modified Ricker function [67] that adjusts expected RS values according to spawner counts on the day a fish reaches the spawning grounds:


$$\overset{⌢}{{RS}_{i}^{\prime }}=\overset{⌢}{RS_{i}}\cdot exp\left(\beta \cdot Nc_{day\;i}\right),$$

where $\overset{⌢}{RS_{i}}$ is the expected RS of individual *i*, predicted by selection on the fitness landscape and representing the maximum $\overset{⌢}{{RS}_{i}^{\prime }}$ value; *Nc*_*day*_ *_i_* is the number of fish alive in the stream on the specific day individual *i* returns to spawn; and *β* represents the magnitude of density dependence. The effect of *β* on population growth was explored (electronic supplementary material, figure S1). Subsequent analyses used *β* = −1 ⨯ 10^−5^, which resulted in approximately stable wild population sizes before hatchery influence. This density dependence module can be interpreted as the net effect of several non-mutually exclusive biological mechanisms that might contribute to density dependence, such as competition for available mates, competition for available spawning habitat or dissolved oxygen depletion. A simplifying assumption of this module is that later returning fish do not affect the RS of earlier returning fish, as might occur in the case of redd superimposition [68].

Although the Ricker function regulated individual RS values, populations could still grow exponentially in simulations with many hatchery fish (electronic supplementary material, figure S1). In natural salmon populations, productivity is constrained by habitat size, limiting the number of redds (nests) in a given gravel area [69]. We therefore set a hard-cap carrying capacity of 4000 individuals, mirroring the size of natural populations in sampled PWS streams [21].

### 2.3. Number of mates module

The number of mates per individual in natural populations is typically limited by factors like competition and behaviour, influencing evolution rates through effects on variance in RS and effective population size. Pacific salmon, for instance, typically have a Poisson-distributed number of mates, with males often having more than females [70,71]. In our simulations, each adult female had a maximum number of mates (*N*_*j*_), drawn from a Poisson distribution with a mean and variance as an initial input parameter (λ_mates_). For each female, *N*_*j*_ temporally overlapping males were randomly drawn as potential mates. Putative pairs of male *i* and female *j* were assigned a weighted probability of being assigned offspring from the product of expected RRS values (*W*_*i,j*_ from equation 2 of May *et al*. [65]). We explored the effects of λ_mates_ on model behaviour in the electronic supplementary materials.

### 2.4. Hatchery straying module

Hatchery-origin fish traits were drawn from a distribution with a different mean return day than natural-origin fish (μ_Return day hatchery_ and μ_Return day natural_), following empirical evidence of later hatchery returns [63]. Mean RLS (μ_RLS_) and the phenotypic variance–covariance matrix (**P**) were assumed to be identical between hatchery and wild fish. Each generation began with an influx of hatchery strays, equal to the rounded product of the number of returning natural-origin adult spawners (Nc_natural_) and the pHOS, an initial input parameter. Hatchery fish were allowed to mate with natural-origin fish if they overlapped temporally and were subject to the same selection regime as natural-origin fish. Thus, the only difference between hatchery and natural-origin fish was in the mean return timing of the two population segments; selection explicitly against hatchery-origin ancestry was not modelled, as it was beyond the aim of this study. Consistent with AHRP terminology, offspring in each generation were considered as ‘natural-origin’ spawners in the subsequent generation, regardless of ancestry.

### 2.5. Sensitivity analyses

Four simulation ‘experiments’ were conducted to examine hatchery straying impacts on population productivity and diversity by varying pHOS and μ_Return day hatchery_. Each unique input parameter combination was considered a ‘simulation,’ whereas ‘experiments’ examined different output metrics. Simulations ran with constant pHOS for 25 generations, mirroring the duration of hatchery operations in PWS [59]. To examine the effects of discontinued hatchery production, pHOS was set to zero after generation 25, and simulations ran for 25 additional generations, totalling 50 per simulation. We performed 1000 replicates per simulation to capture output metric variation. Populations falling below 50 individuals were considered functionally extinct, per May *et al*. [65].

Wherever feasible, input parameters were sourced from empirical AHRP studies from PWS or broader salmon literature. Parameter definitions, values and literature sources are provided in table 2. Here, we provide more context for specific variables used. For both hatchery and wild population segments, initial mean RLS (μ_RLS_) was set equal to 7 days with a variance (σ²_RLS_) of 10 days, following empirical estimates from McMahon [63], who found no significant difference between hatchery and wild RLS. A negative correlation between RLS and return day has been consistently reported for many salmonids including pink salmon in PWS (i.e. McMahon [63]), thus ρ = −0.3 was used. Mean phenotypic optima were held constant at θ_Return day_ = 10, and θ_RLS_ = 7, except in Experiment 4 where θ_Return day_ varied. Interannual variance values were set to σ²_θ Return day_ = 20 days and σ²_θ RLS_ = 1 day, representing moderate and very small environmental variances, respectively, as in May et al. [65]. The strength of stabilizing selection was held constant at ω = 2 phenotypic standard deviations of the fitness landscape, representing moderately strong selection on traits within the range of empirical estimates [75–78]. Despite much larger empirical wild population sizes, Nc_initial_ = 500 individuals were used to aid in computational efficiency in all simulations, with a population-specific carrying capacity of 4000 individuals. Moderate pHOS values of up to 0.3 were used throughout this study, representative of typical streams in PWS (i.e. [11,62]), although values as high as 0.90 have been reported in streams very close to hatchery release sites [62]. Input values for pHOS, μ_Return day natural_, and μ_Return day hatchery_ varied in experiments but were otherwise held constant at values of 0.2, 10 days and 20 days unless otherwise noted (table 1).

**Table 2.** Descriptions, values and literature where values were drawn from for input parameters used in this study. Values were held constant across all simulations unless denoted by asterisk, in which case, see table 1 for values used in different experiments.

input parameter	value	description	value justification
pHOS	0.2*	proportion of hatchery origin spawners to be initiated at the start of each generation	[11,61,62]
μ_Return Day Natural_	10*	initial wild population mean day of return to spawning grounds across a 45 day season; also used as the local phenotypic optimum (θ_Return day_)	[63]
μ_Return Day Hatchery_	20*	population mean day of return to spawning grounds for hatchery fish, used each generation	[63]
μ_RLS_	7	initial wild population mean RLS after returning to spawn, used each generation for hatchery population	[63,72]
σ²_Return Day_	20	population phenotypic variance in return day for both hatchery and wild population segments	[63]
σ²_RLS_	10	population phenotypic variance in RLS for both hatchery and wild population segments	[63]
ρ	−0.3	phenotypic correlation between return day and RLS, for both hatchery and wild population segments	[63,73,74]
**ω**	2	strength of selection (1 **/ω**), measured as the number of phenotypic standard deviations in a stabilizing selection curve	[75–78]
σ²_θ Return day_	20	interannual environmental variance in optimal return day	[65]
σ²_θ RLS_	1	interannual environmental variance in optimal RLS	[65]
λ_mates_	2	mean and variance of the Poisson distribution of maximum number of mates for females	[70,71,79]
β	−1e^−5^	strength of density dependence	electronic supplementary material, figure S1
Nc_initial_	500	census population size of the founder, wild generation (F0)	[65]

*Experiment 1: relative reproductive success*. We explored the impact of temporal segregation between the hatchery and wild groups on RS by varying μ_Return day hatchery_ across four levels (10, 15, 20 and 25 days; figure 1*b*). The wild segment started at μ_Return day hatchery_ = 10, which was also the phenotypic optimum. The wild population was expected to remain near this optimum owing to stabilizing selection while later-returning hatchery segments were further from the optimum. The μ_Return day hatchery_ values represented temporal lags of 0, 5, 10 and 15 days and aligned with observed values in PWS [63]. Using individual fitness outputs, mean RRS values of hatchery fish were estimated [21].

*Experiment 2: productivity*. We assessed how hatchery strays influenced population growth by varying pHOS to 0.0 (negative control), 0.01, 0.1 and 0.3, consistent with reported values for PWS pink salmon streams [11,21,61–63]. For each pHOS value, we varied μ_Return day hatchery_ at 15, 20 and 25 days, representing temporal lags of 5, 10 and 15 days, mirroring Experiment 1. The population size of natural-origin individuals was tracked over generations and compared between simulations.

*Experiment 3: hatchery introgression*. The effect of hatchery strays on natural-origin ancestry was investigated by varying pHOS (values 0.01, 0.1 and 0.3) and μ_Return day hatchery_ (15, 20 and 25 days). In each simulation and time step, we quantified the mean proportion of hatchery-origin ancestry (pHOA) in the natural-origin population. In the first generation, natural-origin fish had a pHOA of 0.0 while strays had pHOA = 1.0; offspring of hatchery × wild crosses had pHOA = 0.5. Conceptually, mean pHOA mirrors the allele proportion from hatchery environments, subject to potential artificial selection (i.e. ‘hatchery load’). Changes in pHOA were monitored over generations and compared between simulations.

*Experiment 4: phenotypic diversity*. We examined hatchery stray impacts on basin-wide phenotypic diversity as a resilience indicator through portfolio effects. ‘Metapopulations’ were simulated, each comprising six spawning streams (i.e. ‘demes’) with varied wild return timing but consistent hatchery stray timing among demes. Demes had unique values for μ_Return day natural_ and θ_Return day_, between days 5 and 20. Four metapopulations were simulated, with different but constant pHOS: 0.0, 0.1, 0.2 or 0.3, while keeping the mean return day of hatchery fish constant at day 13. This design mirrored PWS pink salmon streams, where interpopulation variation in wild return is greater than that of the hatchery return [63]. Assuming no dispersal among demes, we quantified the evolution of mean return day within streams and the coefficient of variation among streams.

## 3. Results

### 3.1. Experiment 1: relative reproductive success

We found that hatchery fish returning later in the season had reduced RRS compared to wild fish (figure 1*c*). For example, hatchery fish returning, on average, 15 days after the average wild fish had RRS = 0.28, 95% CI [0.19–0.36], indicating 72% fewer offspring than natural-origin counterparts. The RRS trends for various mismatch values (i.e. phenotypic lag; figure 1*c*, x-axis) mirrored a normal distribution, aligning with the Gaussian fitness landscape used. RRS did not change after the first few generations, which represented a ‘burn-in’ period (electronic supplementary material, figure S2).

Hatchery fish returning at the phenotypic optimum (i.e. figure 1*b*, dashed line) also had slightly greater RRS (RRS = 1.18, 95% CI [1.16–1.20]), compared to natural-origin counterparts. We attributed this to interannual ecological variation experienced by the natural-origin populations, causing them to continually adapt to shifting optima (centred at day 10 here). After 25 generations, natural fish evolved a mean return day of 10.5, close to the phenotypic optimum of 10, while the hatchery segment averaged 10.1. However, the natural-origin segment had much greater variation in return day over 1000 iterations (σ_Return day natural_ = 4.27) compared with the hatchery group (σ_Return day hatchery_ = 0.03). The hatchery population was centred near the phenotypic optimum more often than the natural population, resulting in increased RRS and reflecting empirical population dynamics if interannual variance in return timing for hatchery-origin fish is less than that of wild populations. However, if variance in return timing is not greater for natural-origin fish, then the RRS values estimated by our model may be slightly inflated owing to this dynamic, as would subsequent rates of demographic and evolutionary change.

### 3.2. Experiment 2: productivity

**Figure 2.**
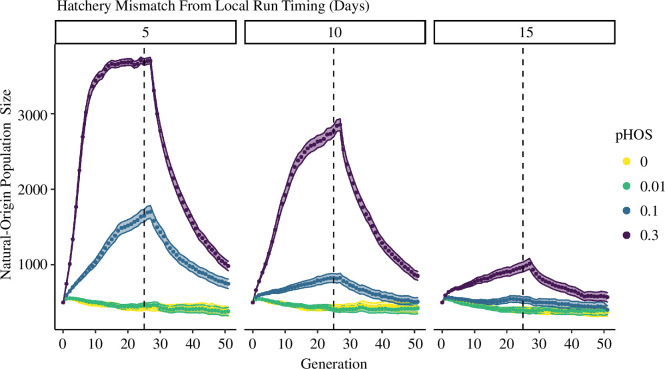
Population sizes of natural-origin fish over 50 generations with varying pHOS (colours) and hatchery fish return timing mismatch (facets). Hatchery production stops after 25 generations (dashed lines). Population sizes are mean estimates from 1000 iterations, with 95% confidence intervals.

Demographic outcomes varied with temporal segregation between hatchery and wild returns, but generally, hatchery inputs increased wild population sizes. Simulations with hatchery populations returning closer to the wild population (closer to the phenotypic optimum) resulted in larger populations (figure 2). The most pronounced example was in simulations with pHOS values of 0.3 (figure 2, purple points); a 15 day phenotypic lag led to population growth from 500 to 970 individuals, 95% CI [906–1037] within 25 generations. In contrast, a 5 day lag saw populations grow to 3683 individuals, 95% CI [3641–3726]. This disparity can be attributed to the RRS differences discussed in Experiment 1, as productivity was determined from individual RS values.

The magnitude of hatchery input also influenced demography. Larger pHOS generally resulted in larger populations, suggesting that hatchery strays contributed to population growth (figure 2). For example, with a 10 day timing mismatch, a pHOS of zero resulted in a population of 436 fish, 95% CI [397–476] after 25 generations, while pHOS = 0.3 saw this number rise to 2771, 95% CI [2693–2849]. However, this relationship also appeared nonlinear (electronic supplementary material, figure S3), as simulations with very small hatchery input (i.e. pHOS = 0.01) resulted in population sizes similar to those with zero hatchery input.

We further examined the demographic outcome of halting hatchery operations after 25 generations of influence. In most cases, population sizes quickly reverted to pre-hatchery levels (figure 2), indicating ceasing hatchery operations may reduce natural population sizes. For example, with a 10 day timing gap and moderate pHOS of 0.1, the population grew from 500 to 826 individuals, 95% CI [767–886] after 25 generations. However, 25 generations after hatchery removal, populations fell to 509 fish, 95% CI [454–564]. Higher spawner abundance reduced individual RS owing to our density dependence module, subsequently reducing productivity.

### 3.3. Experiment 3: hatchery introgression

**Figure 3.**
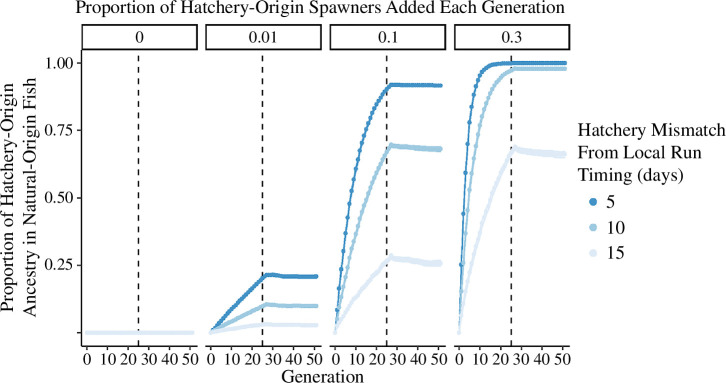
Mean pHOA of natural-origin fish over 50 generations, varying by pHOS (facets) and hatchery fish return timing mismatch (colours). Hatchery production stops after 25 generations (dashed lines). pHOA values are average estimates of 1000 iterations, with 95% confidence intervals.

The pHOA in natural-origin fish increased over time and depended on pHOS and temporal segregation. Increasing pHOS or decreasing temporal segregation led to increased introgression of hatchery ancestry into the natural population. In most scenarios, after 25 generations, pHOA surpassed 25% (figure 3). For instance, in scenarios with minimal pHOS (0.01) and maximum temporal gaps (15 days), introgression was minimal (0.031; 95% CI [0.029–0.033]). However, when pHOS increased to 0.1 or 0.3, pHOA increased to 0.27, 95% CI [0.26–0.27] and 0.67, 95% CI [0.66–068], respectively. Some simulations saw pHOA close to or at 1.0, particularly when pHOS was high (0.3) and temporal segregation was low to moderate (5–10 days).

Ceasing hatchery contributions after generation 25 (figure 3, dashed lines) resulted in little or no decrease in pHOA during the subsequent generations, suggesting natural populations may not recover from hatchery introgression. For example, with moderate pHOS (0.1) and temporal segregation (10 days), pHOA was 0.69, 95% CI [0.68–0.69] after 25 generations. Another 25 generations without hatcheries kept pHOA almost the same, at 0.68, 95% CI [0.67–0.69]. Scenarios with high pHOS showed even less variation. In simulations with high pHOS (0.3) and 5- to 10-day temporal lags, pHOA remained at 0.98 [0.98–0.98] at both the 25th and 50th generation. Notably, selection against hatchery-origin ancestry was not modelled. In real populations, if hatchery-origin alleles were maladaptive in nature, they would likely be selected against, thereby decreasing pHOA.

### 3.4. Experiment 4: phenotypic diversity

**Figure 4.**
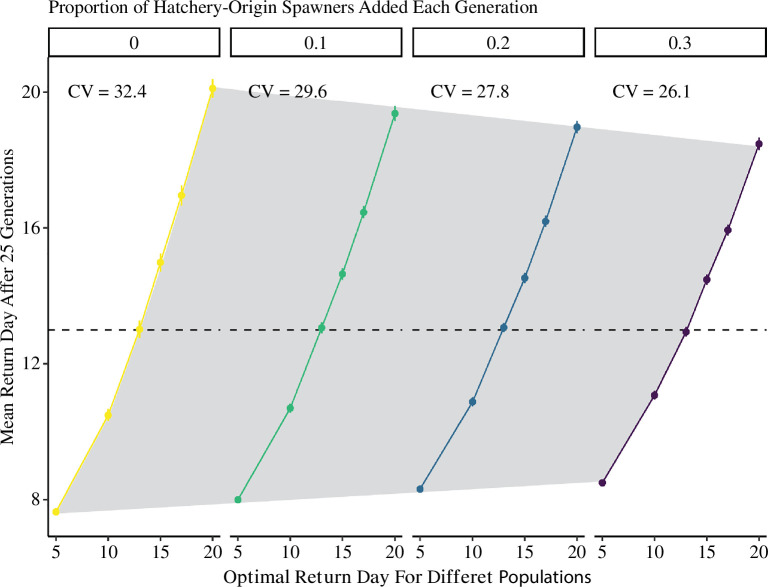
Mean return day of natural-origin individuals after 25 hatchery-influenced generations across four different metapopulations (colours), with varied pHOS (facets). Demes within metapopulations (points) had unique local optima (x-axes), while hatchery-origin fish returned on average on day 13 (dashed line). Values are averaged from 1000 iterations, with 95% confidence intervals.

Increased hatchery influence reduced variation in return timing, indicating hatchery straying may reduce phenotypic diversity in metapopulations. Simulations with zero hatchery influence had a coefficient of variation in mean return day of 32.4 after 25 generations, whereas this value was 26.1 in simulations with pHOS = 0.3 (figure 4). In other words, metapopulations with high pHOS were ~20% less diverse regarding return timing, because demes within metapopulations evolved mean return days closer to day 13, the mean hatchery return day across populations. For example, in simulations with no hatchery influence, demes with local optima equal to day 20 evolved a mean return day of 20.1, 95% CI [19.8–20.4] after 25 generations, but with pHOS = 0.3, these same demes evolved a mean return day of 18.5, 95% CI [18.3–18.7]. However, we also observed that return timing in natural populations quickly evolved back to pre-hatchery distributions upon removal of hatcheries (see also electronic supplementary material, figure S4).

## 4. Discussion

We investigated the impact of hatchery input and phenotypic segregation on the demography and evolution of simulated wild pink salmon populations. Results highlight a trade-off between demographic enhancement and natural population resilience: hatchery introgression increased natural population sizes but diminished phenotypic diversity (here, return timing). In addition, reduced RRS of hatchery strays could be partially explained by phenotypic mismatch in our simulations, and hatchery-origin alleles introgressed into wild populations. While population sizes and phenotypic distributions returned to pre-hatchery levels after hatchery cessation, hatchery-origin ancestry persisted. Previous studies have highlighted a need to investigate specific hatchery–wild interactions [1,6,14], and our simulation framework offers interpretable visualizations of eco-evolutionary dynamics, aiding discussions on hatchery enhancement.

We found a negative correlation between RRS of hatchery fish and temporal segregation from the wild population. The strength of this relationship appeared weaker than in Shedd *et al*. [21], where the observed RRS of hatchery fish was ~0.5, with hatchery fish returning approximately 3–7 days later on average than wild fish. In our simulations, similar RRS was observed when temporal segregation was >10 days. Shedd *et al*. [21] also reported that, even after accounting for return timing and other confounding variables, RRS of hatchery fish was still significantly reduced compared to wild fish. The authors concluded that other causal factors, such as domestication selection on unmeasured traits, may have resulted in maladaptation to the natural environment. The weaker relationship between temporal segregation and RRS in our study may be owing to the fact that, while temporal segregation is likely a contributing mechanism to the reduced RRS of hatchery fish, there are many other factors contributing to fitness-linked differences between hatchery and wild fish that we did not model [1,23,78,80]. Return timing alone could not explain empirically observed reduced RRS of hatchery fish. One unexpected result was in simulations with no phenotypic mismatch; hatchery fish had a slightly greater RRS compared to natural-origin fish. We found this relationship was a result of differences in interannual variation in selection regimes experienced by the natural population, but not by the hatchery population. While this dynamic may be representative of natural systems with interannual variation in fitness optima, RRS values would be inflated if applied to systems where fitness optima vary little. Although we find it unlikely that this mechanism biased the direction of trends, inflated RRS values in our model would result in downstream effects such as faster introgression, demographic growth and evolutionary change. Thus, caution should be taken when interpreting the large magnitude of trends reported here, as they do not account for other important dynamics which may have a stabilizing effect on demographic change. Nevertheless, our framework allowed us to isolate and manipulate a specific phenotypic maladaptation and observe its long-term effects on demography and evolution, which are particularly difficult to quantify in empirical studies.

We anticipated hatchery fish presence to reduce wild stock productivity, presuming annual influxes of hatchery strays would intensify competition and induce phenological shifts [30,44,81,82]. Contrarily, greater hatchery straying increased population sizes, although this effect weakened when hatchery fish were more temporally segregated, had lower breeding values and had fewer mating opportunities with natural-origin fish. While hatchery immigration increased competition and caused natural-origin phenotypes to slightly deviate from the optimum, these factors were minor compared to the large reproductive output of hatchery fish. Thus, in our model, while hatchery fish may produce fewer offspring on average than wild fish, their enhanced reproductive contribution offsets the increased competition and phenotypic mismatch costs. Using a simpler mathematical model, Tufto [83] investigated many of these same dynamics and showed how maladapted immigrants can reduce equilibrium population sizes (i.e. when the magnitude of maladaptation and immigration is large and stabilizing selection is relatively weak); however, this was not the case under most scenarios, as it was not in our simulations. Instead, our results align with empirical observations in PWS and Southeast Alaska showing increased population sizes owing to hatchery presence [9,11,28,57]. Yet, our results contrast those from systems where hatchery supplementation did not increase wild fish abundance, as in the Columbia River basin where factors like habitat degradation may dominate productivity [47]. Importantly, our findings must be contextualized within our model, which assumed hatchery fish were identical to wild fish except for return timing maladaptation. In reality, selection in hatchery settings may alter the expression of hundreds of different genes [84], many of which are unrelated to run timing (i.e. wound healing, disease resistance and juvenile development rate). Additionally, selection in wild populations occurs in a variety of temporally variable freshwater environments, resulting in a diverse fitness landscape [77,85,86]. Therefore, outcomes may differ given maladaptation(s) in other heritable traits and stream environments, emphasizing complexities of hatchery–wild interactions and population adaptations to local habitats. Still, the extent of maladaptation must be substantial to eclipse the demographic boost from hatcheries to natural populations below carrying capacity.

In our simulations, hatchery-origin immigration increased both the number and within-population phenotypic variation of spawners above equilibrium levels; following the termination of hatchery releases, population sizes declined towards initial equilibrium sizes. The return to equilibrium following hatchery cessation can be seen as the result of density dependence acting on the inflated number of spawners and stabilizing selection acting on the inflated phenotypic variation. This demographic boost and subsequent return towards equilibrium are most akin to dynamics seen in conservation hatchery settings, where a primary goal of hatchery production is to reduce the depensatory effects of low spawning numbers [87] and boost population sizes towards local carrying capacities [88]. However, there are few comparable empirical studies that quantify demographic changes after hatcheries reduce releases or cease altogether [89,90]. One such study on coho salmon in the Salmon River in Oregon found that, unlike our results, hatchery termination was associated with increased natural-origin adult abundance and spawning season duration, attributable to greater survival of wild fish at-sea (i.e. broad spawning season duration may have provided better phenotypic match with environment; [91]). However, this example in coho salmon was also associated with a distinct resurgence of an estuary-rearing juvenile ecotype. This comparison highlights a limitation of our model; we did not simulate life stages, life history variation (other than spawn timing), or account for selection and density-dependent effects other than those experienced on the spawning grounds. Importantly, the timing and relative intensity of density dependence and selection during the life cycle can differentially affect the magnitude and direction of demographic changes [45]. While focusing our simulations on spawning ground dynamics allowed us to better understand specific components of hatchery impacts, results should be applied with caution when drawing conclusions about overall demographic impacts or other species with greater life history variation.

Our analyses demonstrated a rise in pHOA in natural-origin populations across generations, more so with high pHOS and low temporal segregation, demonstrating the potential impact of hatchery strays on wild genetic composition. While some studies hypothesize that maladapted hatchery lineages would be swiftly purged from wild populations [1,43,45,92], we found indications of enduring genetic and adaptive shifts. Even after removing hatcheries, hatchery-origin ancestry persisted, consistent with previous findings of the persistence of hatchery-origin alleles in wild genomes [23,93–95]. Our model excluded gene flow from less hatchery-influenced areas (e.g. eastern PWS; [11]), despite substantial straying of wild pink salmon (i.e. 2–25%; [96,97]). Gene flow from such less-affected regions might influence local allele frequencies over time [98]. Hatchery introgression is also not inherently detrimental and can, in some cases (e.g. small, inbred populations) increase genetic diversity [99]. Furthermore, PWS pink salmon maintained significant genetic structure despite prolonged hatchery production, mirroring diversity in less-affected areas [100,101]. Maintenance of this diversity in the presence of large numbers of both hatchery and wild strays supports the model results that selection can rapidly change the expression and variation of fitness-related traits. Nonetheless, domestication selection still poses risks in pink salmon hatcheries, despite minimal hatchery residence time compared to other species, and particularly in populations with large effective sizes owing to the increased effectiveness of selection. We also note that selection acts on specific alleles, and patterns of hybridization and selection may return population allele frequencies of loci under selection to pre-hatchery levels while pHOS remains relatively stable. Yet, the potential for hatchery-origin alleles to enter wild gene pools is still concerning, as these traits may have no effect on fitness until environmental shifts reveal negative fitness impacts. Future research should continue to investigate long-term effects of hatcheries on wild populations, particularly the potential for latent genetic changes with deleterious impacts on population fitness. Controlled laboratory experiments examining genotype-by-environment interactions in hatchery and wild fish could further add to our understanding of the potential effects of hatchery strays on the adaptive potential of wild populations.

High pHOS values reduced return timing variation among populations, resulting in phenotypic homogenization. However, our model assumed no dispersal between streams, which would likely intensify this effect. Such reduced phenotypic diversity aligns with previous findings in other species [26,28,102,103] and offers a mechanistic explanation for observed reductions in phenotypic diversity in supplemented populations. Reduced diversity may reduce resilience owing to eroded portfolio effects [35]. Given strong genetic controls over phenological traits in salmon [49,104,105], potentially by large effect loci [5,41,106–108], genetic homogenization may impact long-term adaptability and viability of these populations [24,40,109]. Importantly, genetic homogenization compounded with demographic synchrony may greatly influence portfolio effects [40]. However, post-hatchery removal, we found phenotypic diversity quickly recovered to original levels, as local fitness landscapes favoured historical return timing distributions. Additionally, the environment strongly affects pink salmon return timing, suggesting parallel evolution among populations and demonstrating divergent selection on phenology among habitats [50]. Nevertheless, proactive measures to reduce phenotypic and genetic diversity loss are preferred. Overall, findings underscore the importance of management practices that monitor and preserve wild diversity across large spatial and temporal scales.

It is crucial to acknowledge model assumptions when considering applications to management. For example, we posited an association between pHOS and hatchery release sizes, suggesting larger releases result in higher pHOS. However, pHOS is also shaped by factors like distance between hatcheries and wild streams [110], hatchery conditions and release practices [12], recipient wild population size [111] and fisheries management. Factors like broodstock genetic variation and environmental conditions may affect temporal segregation. The effects of domestication selection on at-sea or juvenile mortality might further obscure these relationships. PWS pink salmon exemplify these complexities, as pHOS or temporal dynamics may be decoupled from hatchery release numbers owing to spatial and temporal targeting of hatchery fish by fisheries and the differing locations and practices of several different hatcheries. Here, we assumed no other factors influenced fitness or productivity, like juvenile survival [14]. We focused on temporal spawning dynamics, with no divergence in selective regimes or density dependence across other life stages or traits, the magnitude and timing of which can impact demographic outcomes [45]. Demography and evolution in real populations may be minimally affected by pHOS and temporal segregation. Furthermore, our quantitative genetic framework assumed return timing was controlled by many loci of small effect, whereas recent research is showing that large-effect loci may explain substantial variation in this trait [108]. We also did not consider the spatial genetic structure between hatchery and wild fish. In PWS, strays tend to spawn higher upstream [21], possibly owing to original broodstock selection [54], and salinity boundaries drive spatial assortative mating [64]. Increased spatial segregation might reduce introgression similar to temporal segregation. Yet, complexities associated with population dynamics are outside the scope of this study, and it is imperative to avoid simplistic interpretations of findings. Our model mainly reflects productivity changes from specific temporal dynamics, highlighting the need for a comprehensive assessment of interacting variables before making management decisions.

In conclusion, we applied a novel tool [65] to examine the interplay among hatchery practices, population demographics, evolution and phenotypic diversity in PWS pink salmon. Our results underscore the potential demographic benefits of hatcheries to wild populations while highlighting risks for associated negative outcomes like genetic and phenotypic homogenization. These findings call for careful management strategies that balance demographic enhancement with conservation priorities. While recognizing our model’s constraints, we advocate for future research on eco-evolutionary interactions between hatchery and wild salmon, especially potential latent genetic changes impacting long-term population fitness. While wild salmon populations are diverse and resilient, it is crucial to consider how management strategies might modulate mechanisms underlying pHOS and temporal segregation to mitigate for risks of introgression and homogenization. Our model may be extended to different hatchery dynamics, eco-evolutionary questions in other structured systems and toward guiding sustainable management practices.

## Data Availability

Data and relevant code for this research work are stored in GitHub [112] and have been archived within the Zenodo repository [113]. Supplementary material is available online [114].
